# Impact of cardiac and respiratory motion on the 3D accuracy of image-guided interventions on monoplane systems

**DOI:** 10.1007/s11548-023-02998-9

**Published:** 2023-07-21

**Authors:** Dagmar Bertsche, Patrick Metze, Leonhard-Moritz Schneider, Ina Vernikouskaya, Volker Rasche

**Affiliations:** Department of Internal Medicine II, Ulm University Medical Center, Ulm, Germany

**Keywords:** Image-guided intervention, 3D accuracy, Motion-induced accuracy-effects, Monoplane system

## Abstract

**Purpose:**

Image-guided intervention (IGI) systems have the potential to increase the efficiency in interventional cardiology but face limitations from motion. Even though motion compensation approaches have been proposed, the resulting accuracy has rarely been quantified using in vivo data. The purpose of this study is to investigate the potential benefit of motion-compensation in IGS systems.

**Methods:**

Patients scheduled for left atrial appendage closure (LAAc) underwent pre- and postprocedural non-contrast-enhanced cardiac magnetic resonance imaging (CMR). According to the clinical standard, the final position of the occluder device was routinely documented using x-ray fluoroscopy (XR). The accuracy of the IGI system was assessed retrospectively based on the distance of the 3D device marker location derived from the periprocedural XR data and the respective location as identified in the postprocedural CMR data.

**Results:**

The assessment of the motion-compensation depending accuracy was possible based on the patient data. With motion synchronization, the measured accuracy of the IGI system resulted similar to the estimated accuracy, with almost negligible distances of the device marker positions identified in CMR and XR. Neglection of the cardiac and/or respiratory phase significantly increased the mean distances, with respiratory motion mainly reducing the accuracy with rather low impact on the precision, whereas cardiac motion decreased the accuracy and the precision of the image guidance.

**Conclusions:**

In the presented work, the accuracy of the IGI system could be assessed based on in vivo data. Motion consideration clearly showed the potential to increase the accuracy in IGI systems. Where the general decrease in accuracy in non-motion-synchronized data did not come unexpected, a clear difference between cardiac and respiratory motion-induced errors was observed for LAAc data. Since sedation and intervention location close to the large vessels likely impacts the respiratory motion contribution, an intervention-specific accuracy analysis may be useful for other interventions.

## Introduction

Over the last years, image-guided intervention (IGI) systems have gained interest in interventional cardiology. The fusion of different imaging modalities has been proven to save procedure time and radiation dose [1–3] with the potential to improve patient outcome, especially for the increasingly complex percutaneous procedures in structural heart disease. Even though the fusion of periprocedural imaging modalities such as x-ray fluoroscopy (XR) and transoesophageal echocardiography (TEE) is increasingly applied [4–6], the additional integration of patient-specific anatomic and functional data, e.g., derived from preprocedural imaging such as computed tomography (CTA) and cardiac magnetic resonance imaging (CMR), appears as an attractive adjunct for providing accurate three-dimensional (3D) information for navigation and documentation [7–10].

The applicability of IGI systems, however, is restricted by their reliability and accuracy. Especially in interventional cardiology accuracy issues rise due to likely differences between pre- and periprocedural soft tissue anatomy, missing landmarks for straightforward co-registration, and motion of the target structure. Thus, in most reported interventional cardiac applications, IGI systems are commonly used for providing adjunct 3D information but the application for advanced navigation has so far not entered daily clinical routine [11].

Improved co-registration techniques have been presented for 3D-3D as well as for 2D-3D co-registration [12–14], and motion compensation approaches have been introduced mainly focusing on the compensation of respiratory motion [15–17]. As desired accuracy of an IGI system, a minimum of 5 mm has been reported for endovascular and cardiac procedures [18, 19]. Furthermore, the distinction between intervention-specific clinically-imposed accuracy restrictions and the IGI system’s limitations was recommended [20, 21]. Where the general accuracy of the co-registration between pre- and periprocedural imaging has been proven sufficient in phantom studies the impact of cardiac or respiratory motion on the accuracy of IGI systems has rarely been assessed [14, 22, 23].

The purpose of this study is to assess the limitations of fusing pre- and periprocedural image data during intervention guidance based on in vivo data, and to quantify the impact of respiratory and cardiac motion on the achievable 3D accuracy. The analysis is based on the assessment of the 3D location of the marker of the deployed left atrial appendage closure (LAAc) device, which is documented in the fused preprocedural CMR data during the intervention and compared to the real location identified in the postprocedural CMR.

## Methods

The accuracy of the 3D location of specific device markers identified in preprocedural CMR data based on periprocedural XR data has been quantified in routinely acquired patient data obtained during percutaneous left atrial appendage closures. After co-registration of preprocedural CMR and XR data, the 3D locations of the radio opaque marker of the implanted occluder device (Watchman FLX™, Boston Scientific Corporation, Marlborough, MA, USA) were identified in XR data and their 3D location derived and marked in the preprocedural CMR for different cardiac and respiratory phases. For accuracy assessment, an additional CMR was acquired postprocedurally and co-registered to the preprocedural CMR and hence XR. The distance between the XR-derived 3D marker position and the respective marker location identified in the co-registered postprocedural CMR was used for cardiac and respiratory phase-dependent accuracy assessment.

Six patients undergoing LAAc were included in this study. Retrospectively, the XR data routinely acquired during the procedure were evaluated. One of the patients had to be excluded as the validation XR-run was too short with shallow respiration, and hence did not allow the distinction of different respiratory phases.

The analysis was performed in compliance with the ethical guidelines of the Declaration of Helsinki from 1975 and was approved by the local ethical committee. The procedure was performed according to the current clinical standards, and the retrospective data analysis has no impact whatsoever on the procedure guidance and XR data acquired. All patients provided written informed consent prior to the procedure.

### Image data acquisition

#### Magnetic resonance imaging

On the day before the LAAc and 4–6 weeks after the procedure, the patients underwent CMR at a 3.0 T (Achieva 3.0 T, dStream, R5.6, Philips Medical Systems B.V., Best, The Netherlands). All data were acquired with a respiratory navigated (3 mm acceptance window) non-contrast enhanced 2-point 3D Dixon sequence [24] during expiration and at 30–40% RR-interval with an isotropic spatial resolution of 1.5^3^ mm^3^ reconstructed at 1.3^3^ mm^3^.

#### X-ray fluoroscopy

In adjunction to transoesophageal echocardiography for guiding the transseptal puncture and LAA dimension assessment, XR was periprocedural applied to guide the procedure, measure the LAA dimensions, and validate the correct positioning of the occluder. XR data were acquired on a monoplane XR system (Allura Clarity, Philips Medical Systems, Best, The Netherlands) at different clinically indicated procedure steps including: prior to the transseptal puncture (anterior–posterior angulation), contrast agent-enhanced after advancing the catheter into the LAA (two angulations for LAA dimension assessment), and after occluder deployment for validation (implantation plane angulation and angulation differing by at least 30°).

The cardiac phase of each frame of a single XR run was determined based on the simultaneously recorded ECG. The respective respiratory phase was identified by manual tracking of the diaphragm (if visible) or a clearly visible lung structure. The respiration tracking result was low-pass filtered with a Savitzky-Golay-Filter. To comply with the CMR data acquisition, an acceptance window in the iso-center of 3 mm was used for classifying frames in expiration or inspiration.

### Co-registration

The preprocedural CMR data were co-registered with the XR-system geometry and the post-procedural CMR data were co-registered to the preprocedural CMR data thus enabling direct comparison of 3D locations between XR and pre- and post-CMR.

#### Co-registration: pre- to periprocedural (CMR to XR)

**Fig. 1:**
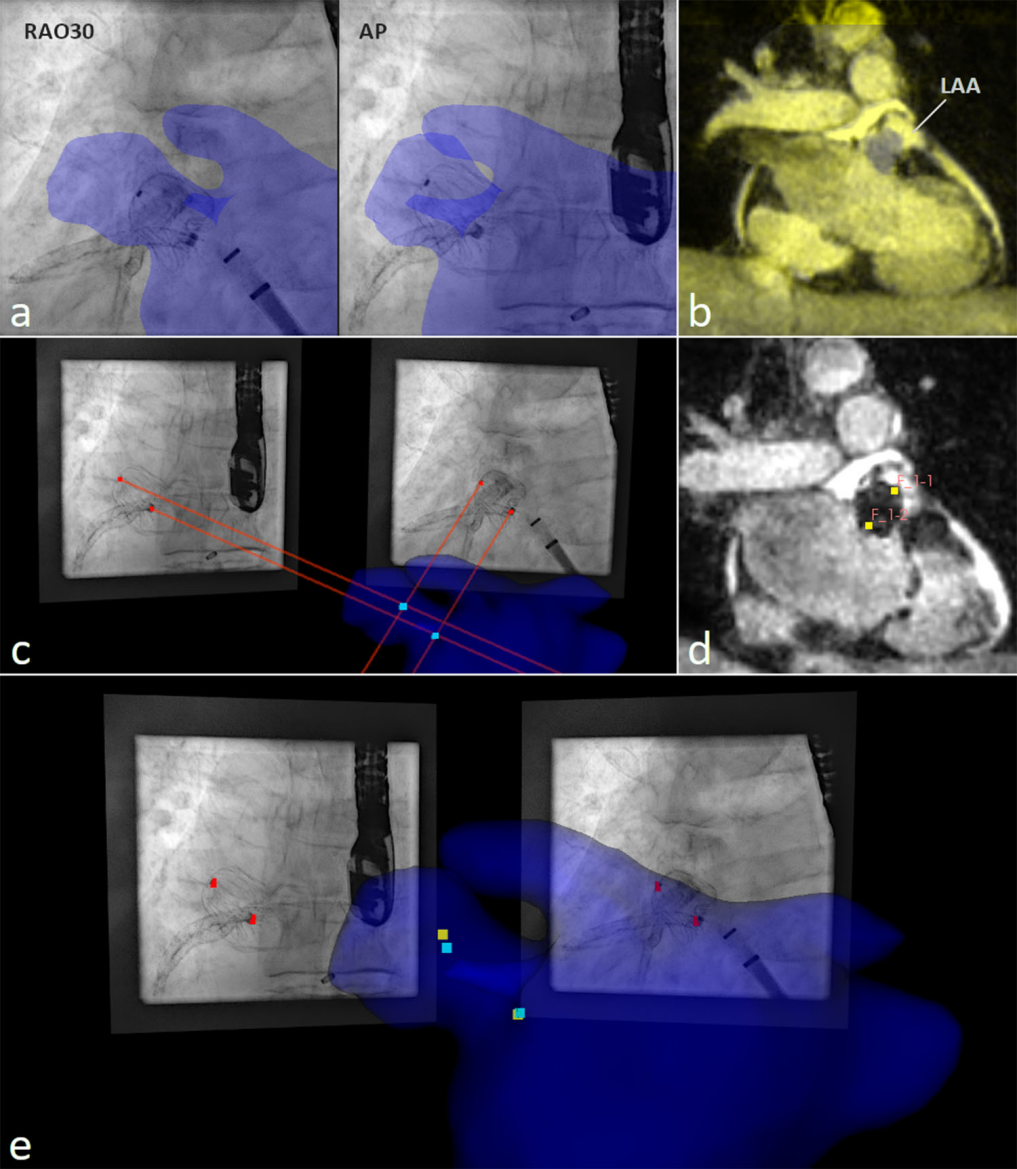
Validation approach: **a** co-registration of the LA including the LAA (blue structure) segmented from preprocedural CMR to periprocedural XR-runs; **b** co-registration of the postprocedural CMR (yellow scaled) to the preprocedural CMR (grayscaled); **c** device marker localized in 3D (${P}_{i}^{{\mathrm{XR}}_{\mathrm{pair}}}$, cyan marker) from marker positions identified in two XR projections (red marker) and their projection lines (red lines) relative to the co-registered LA segmentation (blue structure); **d** device marker identified in postprocedural CMR (${P}_{i}^{{\mathrm{CMR}}}$, yellow marker); **e** XR-derived 3D marker positions (${P}_{i}^{{\mathrm{XR}}_{\mathrm{pair}}}$, cyan marker) reconstructed from marker identified in XR (red marker) to device marker positions identified in postprocedural CMR (${P}_{i}^{{\mathrm{CMR}}}$, yellow marker) visualized with the preprocedural LA segmentation (blue structure)

First, the aorta, the right atrium, the vena cava, and the left atrium including the left atrial appendage were segmented from the preprocedural CMR using the open-source tool 3DSlicer (www.slicer.org, [25]). From the resulting segmentation, manual rigid body co-registration with the XR system was performed using the open-source tool 3D-XGuide [22]. Thereby, initial co-registration was done based on XR-runs in anterior–posterior (AP) and left anterior oblique (LAO40) angulation by aligning the aorta and right atrium to their projection in XR as previously suggested [9]. Refinement of the co-registration was done based on the contrast agent-filled LAA in the runs acquired for dimension measurements. As the CMR data were acquired at a certain cardiac- and respiratory phase, frames of the XR-run in the respective phases (30–40% RR-interval and expiration) were selected for accurate final adjustments (Fig. 1a).

#### Co-registration: post- to preprocedural CMR

An initial rigid-body transformation was performed manually using 3DSlicer. Thereafter, an automated affine transformation was generated with the BRAINSFit toolkit integrated into 3DSlicer [26] (Fig. 1b).

### Accuracy assessment

The accuracy was assessed from the distance of the device marker locations identified in the co-registered peri- and postprocedural data. In all cases, the accuracy was assessed for data obtained in the same cardiac- and respiratory phase (motion-synchronized) and for data obtained in different phases to quantify the impact of the different motion components on the final accuracy.

#### Motion-synchronized 3D accuracy

Based on the frame pair comprising the frames of the validation XR in corresponding cardiac and respiratory phase, the two device markers were identified manually and the respective 3D location (${P}_{i}^{{\mathrm{XR}}_{\mathrm{pair}}})$ calculated using the well-known camera models as previously described [27, 28]. In detail, the Nadir points on the projection lines of the identified marker at the smallest distance between these lines was determined. Ideally, the two projections lines would intersect at${P}_{i}^{{\mathrm{XR}}_{\mathrm{pair}}}$, but in real world data the mid-point between the two Nadir points had to be defined as $P_{i}^{{{\text{XR}}_{{{\text{pair}}}}^{{}} }}$, as the projection lines intersect only approximately (Fig. 1c). Since the XR frames in the different angulations were acquired during subsequent runs, the accuracy of the XR-derived 3D localization of $P_{i}^{{{\text{XR}}_{{{\text{pair}}}}^{{}} }}$ was first assessed by re-projecting ${P}_{i}^{{\mathrm{XR}}_{\mathrm{pair}}}$ onto the individual XR frames and calculating the respective distance to the identified marker locations in the XR image plane. For the assessment of the motion-synchronized 3D accuracy, the distance between the $P_{i}^{{{\text{XR}}_{{{\text{pair}}}}^{{}} }}$ and the positions of the marker identified in the co-registered postprocedural CMR (${P}_{i}^{\mathrm{CMR}}$) was calculated (Fig. 1d, e). The resulting distances are reported as the averaged Euclidean distance (AED) with standard deviation (± SD).

#### Impact of motion on the 3D accuracy

**Fig. 2:**
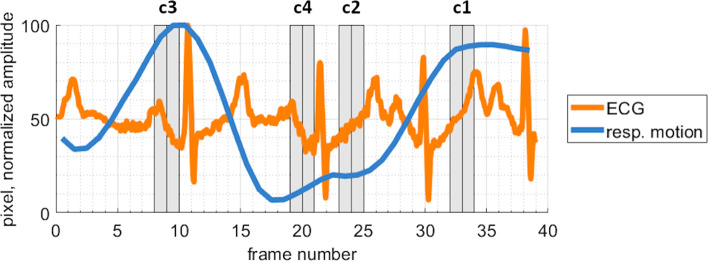
Exemplified frame classification of a XR-run into the categories (c1) expiration; 30–40% RR-interval; (c2) inspiration; 30–40% RR-interval; (c3) expiration; 80–90% RR-interval; (c4) inspiration; 80–90% RR-interval determined based on ECG and tracked respiratory motion

To investigate the influence of heart beat and respiration on the accuracy, the XR frames were categorized into four different categories (Fig. 2) based on the assigned cardiac and respiratory phase: (c1) expiration; 30–40% RR-interval; (c2) inspiration; 30–40% RR-interval; (c3) expiration; 80–90% RR-interval; (c4) inspiration; 80–90% RR-interval. Due to the only short duration of the 2nd angulation XR run after deployment, the classification was only done for the first angulation. As the duration of the XR run and the respective cardiac/respiratory phases varies, different numbers of frames were assigned to each category (15 samples in (c1), 7 samples in (c2), 17 samples in (c3), 9 samples in (c4)). The Nadir point of the co-registered postprocedural ${P}_{i}^{\mathrm{CMR}}$ on the projection line of the 2D-XR-derived marker position, equivalent to the closest point on the projection line to ${P}_{i}^{\mathrm{CMR}}$, was determined as the 3D marker position ${P}_{i}^{{\mathrm{XR}}_{\mathrm{single}}}$ derived from a single XR-projection. The AED ± SD between ${P}_{i}^{{\mathrm{XR}}_{\mathrm{single}}}$ and the ${P}_{i}^{\mathrm{CMR}}$ was used as an accuracy measure.

#### Statistics

Category c1 corresponds to the same cardiac and respiratory phase as used during CMR and is considered motion-synchronized. After testing for normal distribution of the samples using the Shapiro–Wilk test, the significance of the differences between c1 and c2–4 was tested applying Welch’s test. A *p*-value < 0.05 was considered significant.

### Accuracy estimation

Assuming an almost ideal co-registration and negligible patient motion between the subsequently acquired XR validation runs, the estimated accuracy is governed by the accuracy of the manual identification of the marker points in XR and CMR. The accuracy of the marker identification is based on the underlying spatial image resolution. Assuming a maximal inaccuracy of the half diagonal of the respective voxel (XR: 2D, CMR: 3D), the maximum error was estimated by ${\varepsilon }_{2\mathrm{DXR}}{\prime}$= 0.2 mm, respectively, ${\varepsilon }_{\mathrm{CMR}}{\prime}$= 1.1 mm. For the accuracy estimation of 3D localization from two XR projections, ${\varepsilon }_{{2\mathrm{DXR}}}{\prime}$ projected to the iso-center ${\varepsilon }_{{2\mathrm{DXR}}_{\mathrm{iso}}}{\prime}$ = 0.1 mm and the intrinsic inaccuracy of the XR geometry system of approximately $\varepsilon _{{{\text{XR}}_{{{\text{geo}}}} }} ^{\prime }$= 0.2 mm [29] were taken into account, resulting in ${\varepsilon }_{{3\mathrm{DXR}}}^{\prime}$_≈_ $\sqrt{{\varepsilon }_{{2\mathrm{DXR}}_{\mathrm{iso}}}^{{\prime}2}+ {\varepsilon }_{{\mathrm{XR}}_{\mathrm{geo}}}^{{\prime}2}}$ = 0.2 mm. ${\varepsilon }_{{3\mathrm{DXR}}}^{\mathrm{^{\prime}}}$ re-projected onto the image plane resulted in ${\varepsilon }_{{2\mathrm{DXR}}_{\mathrm{proj}}}{\prime}$= 0.3 mm. Accounting XR- and CMR-derived error sources, a total 3D accuracy estimation of ${\varepsilon }_{\mathrm{tot}}{\prime}$_≈_ $\sqrt{{\varepsilon }_{{3\mathrm{DXR}}}^{{\prime}2}+ {\varepsilon \mathrm{^{\prime}}}_{\mathrm{CMR}}^{2}}$ = 1.1 mm was considered. 3D localizing from only a single monoplane projection reduces the accuracy but can be neglected as it does not affect the total accuracy estimation calculated from the 3D localization from two projections.

## Results

The assessment of the resulting accuracy depending on the motion consideration employed was possible based on the patient data. Device marker were identifiable in XR and postprocedural CMR data. Based on the non-contrast-enhanced CMR, the segmentation of structures relevant for LAAc was successful. 3D-3D co-registration between pre- and postprocedural CMR as well as 2D-3D co-registration between preprocedural CMR and XR was visually correct.

The device markers ${P}_{i}^{{\mathrm{XR}}_{\mathrm{pair}}}$ re-projected onto the respective image plane deviated on average by 0.5 ± 0.5 mm from the initial marker position identified in the XR images. Thereby the estimated accuracy of XR-derived 3D localization of 0.4 mm was slightly exceeded. The largest inaccuracy of the position of ${P}_{i}^{{\mathrm{XR}}_{\mathrm{pair}}}$ was observed for the 3D localization based on two projections with the smallest angular difference (Table 1).

**Table 1:** AED between ${P}_{i}^{{\mathrm{XR}}_{\mathrm{pair}}}$ re-projected onto the XR image plane and marker positions identified initially in the XR image plane (${\varepsilon }_{{2\mathrm{DXR}}_{\mathrm{proj}}}$) depending on the angular difference between the projections used for 3D localization and motion-synchronized 3D accuracy assessed by the AED between ${P}_{i}^{{\mathrm{XR}}_{\mathrm{pair}}}$ and respective co-registered ${P}_{i}^{{\mathrm{CMR}}}$ (${\varepsilon }_{{3\mathrm{DXR}}}$)

ID	Angulation difference in °	${\upvarepsilon }_{{2\mathrm{DXR}}_{\mathrm{proj}}}$ in mm AED (SD)	${\upvarepsilon }_{{3\mathrm{DXR}}}$ in mm AED (SD)
1	30	1.5 (0.2)	1.7 (0.5)
2	40	0.4 (0.0)	4.3 (0.9)
3	80	0.5 (0.2)	1.6 (0.6)
4	72	0.1 (0.0)	2.4 (1.4)
5	85	0.2 (0.1)	1.6 (0.4)
**Ø**		**0.5 (0.5)**	**2.3 (1.0)**

The motion-synchronized $P_{{}}^{{{\text{XR}}_{{{\text{pair}}}}^{{}} }}$ differed from the respective co-registered ${P}_{i}^{{\mathrm{CMR}}}$ on average by 2.3  ± 1.0 mm, which is again slightly above the theoretical limit of 1.1 mm (Table 1).

**Fig. 3:**
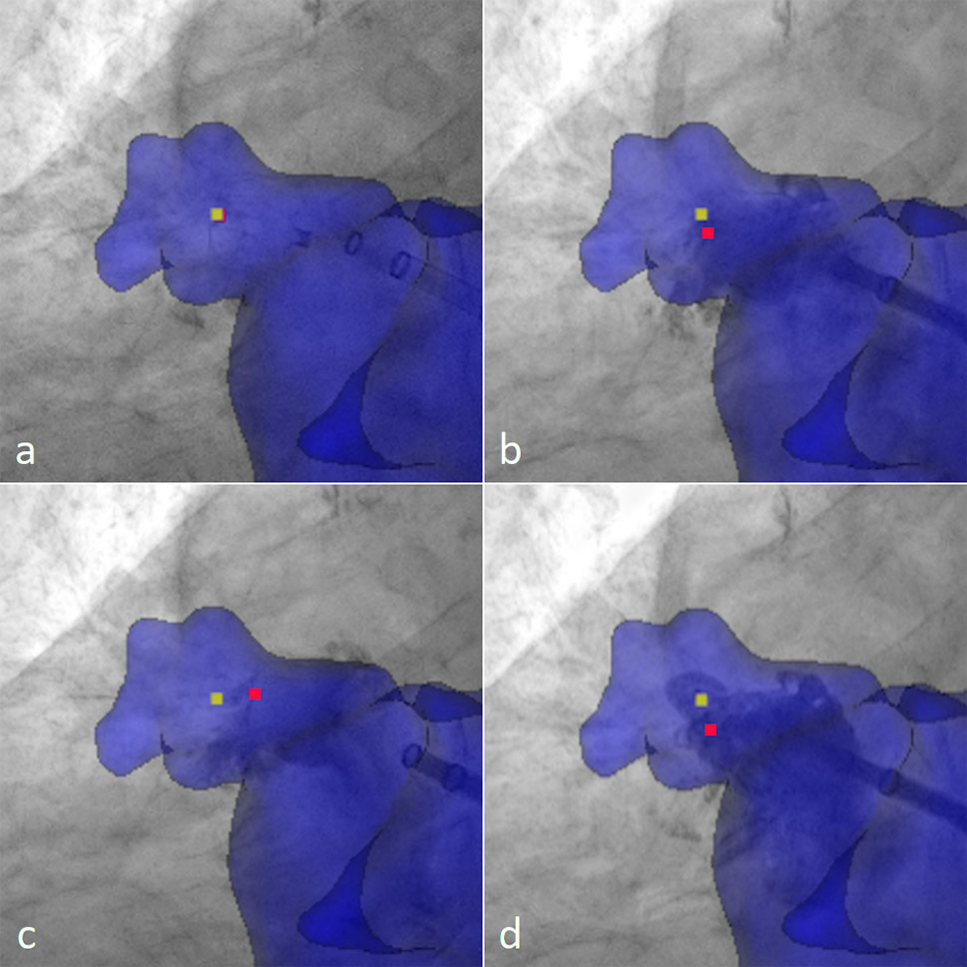
The effect of motion on the accuracy of image guidance. Preprocedural CMR segmentation (blue) and postprocedural marker (${P}_{i}^{{\mathrm{CMR}}}$, yellow marker) acquired in expiration and 30%–40% RR-interval co-registered and superimposed to the periprocedural XR data with identified device marker (${P}_{i}^{{\mathrm{XR}}_{\mathrm{single}}}$, red marker) in different heart and respiratory phases: **a** expiration and 30–40% RR-interval (c1, motion-synchronized, marker overlap), **b** inspiration and 30–40% RR-interval (c2), **c** expiration and 80–90% RR-interval (c3), **d** inspiration and 80–90% RR-interval (c4)

**Fig. 4:**
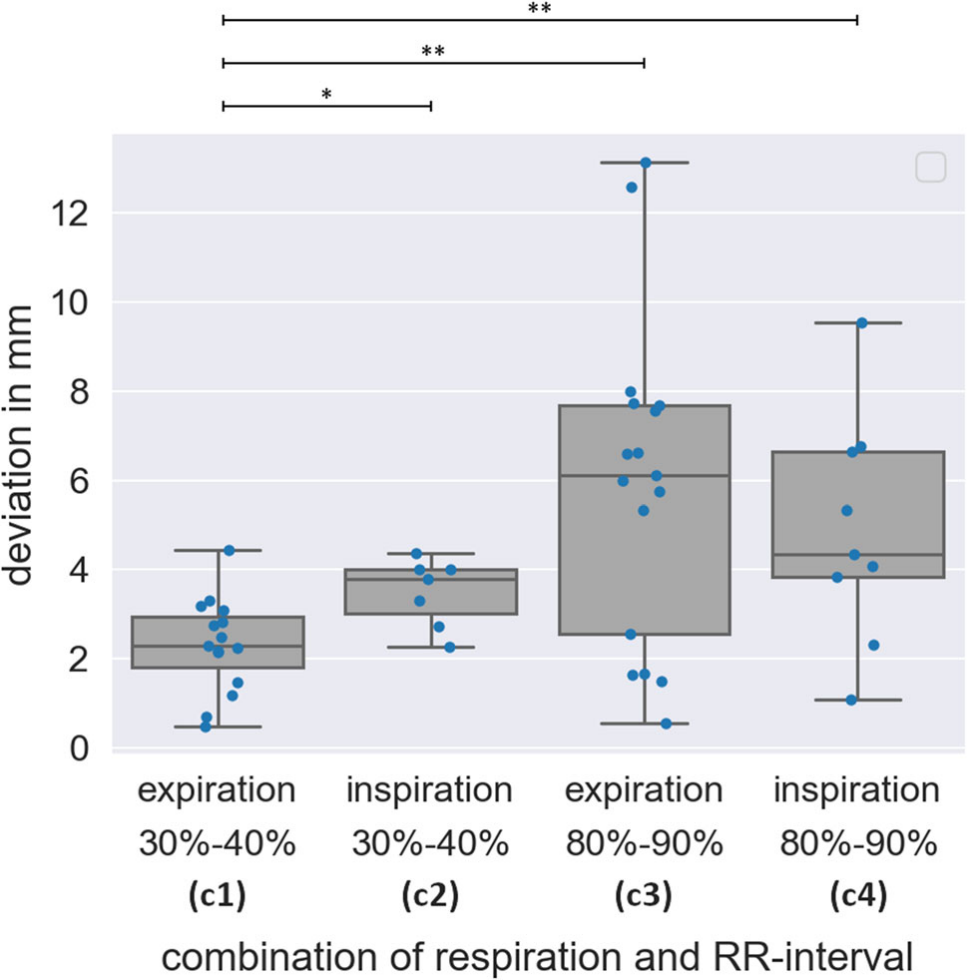
Effect of motion on the accuracy assessed by the distance of ${P}_{i}^{{\mathrm{CMR}}}$ to ${P}_{i}^{{\mathrm{XR}}_{\mathrm{single}}}$ for different cardiac and respiratory phases. Motion-synchronized image fusion (c1: expiration, 30–40% RR-interval) achieved significantly (*p < 0.05;**p < 0.01) superior accuracy than non-motion-synchronized image fusion (c2–c4)

Figure 3 shows the effect of respiration- and cardiac-induced motion on the accuracy of image fusion. On average, the distance (m) of ${P}_{i}^{{\mathrm{XR}}_{\mathrm{single}}}$ of category c1 (motion-synchronized) to the respective co-registered $P_{i}^{{{\text{CMR}}_{{}}^{{}} }}$ (m = 2.3 mm ± 1.0 mm) was slightly larger than the estimated accuracy ${\varepsilon }_{\mathrm{tot}}{\prime}$ and equal to the distance observed for ${P}_{i}^{{\mathrm{XR}}_{\mathrm{pair}}}$ of the corresponding category. The distances of ${P}_{i}^{{\mathrm{CMR}}}$ to ${P}_{i}^{{\mathrm{XR}}_{\mathrm{single}}}$ of categories c2 (*m* = 3.5 ± 0.7 mm; *p* < 0.05), c3 (*m* = 5.9 ± 3.5 mm; *p* < 0.01), and c4 (m = 4.9  ± 2.4 mm; *p* < 0.01) significantly exceeded the distances of ${P}_{i}^{{\mathrm{CMR}}}$ to ${P}_{i}^{{\mathrm{XR}}_{\mathrm{single}}}$ of category c1. As visualized in Fig. 4, the distances resulting from XR data obtained during atrial systole (c3, c4) showed larger mean values and interquartile ranges as during atrial diastole (c1, c2). For XR data obtained during atrial diastole, the resulting distances were on average larger in inspiration than in expiration, but showed similar interquartile ranges. Minimal distances were obtained for the motion-synchronized data (c1).

## Discussion

Even though image-based interventional guidance systems are frequently applied during different applications, the possible accuracy in organs impacted by respiratory and cardiac motion has not been investigated in vivo. In the presented study, we used data obtained during left arterial appendix closure (LAAc) procedures to initially investigate the possible accuracy of fusing preprocedural anatomic data with periprocedural XR fluoroscopy in a small number of patients. In the LAAc procedure, the 3D locations of the radio opaque closure device marker were calculated from two XR views and superimposed onto the co-registered 3D CMR data. The anatomic locations of the markers were validated in a postprocedural 3D CMR and compared to the 3D XR-derived locations after co-registration of the pre- and postprocedural CMR data.

Based on the patient data, the impact of the organ motion on the final accuracy could be assessed. As expected, the accuracy resulting from the fusion of the pre- and periprocedural data matching the respiratory and cardiac phase of the preprocedural 3D CMR yielded the smallest errors. Even though the achieved accuracy does not yet match the theoretical estimated limit, the previously reported minimal accuracy for endovascular and cardiac interventions of 5 mm [18, 21] could be clearly exceeded. The mismatch with the theoretical accuracy might be explained by additional unknown error sources. Here especially the angulation-dependent bending of the C-arm, not perfect co-registration between the pre- and periprocedural imaging, the subsequent acquisition of the XR views on a monoplane system, and, very likely most important, repositioning of the patient between the preprocedural CMR and the LAAc procedure impact the achievable accuracy.

Choosing XR data from different cardiac and/or respiratory phases yielded a significant increase of the resulting error in the calculated anatomic position of the marker. Where the general decrease in accuracy did not come unexpected, a clear difference between cardiac and respiratory motion-induced errors was observed. XR data with motion-synchronized cardiac but differing respiratory phase mainly increased the mean error with still rather low variance, thus indicating a predominant shift of the anatomic location. XR data from different cardiac phases, however, revealed large variance, which indicates non-predictable shifts of the resulting anatomic location. The huge impact of the cardiac motion may be specific for LAAc as the atrium contracts during atrial systole causing a substantial motion to the LAA. As previously shown [30], the respiratory-induced motion amplitude varies between different cardiac locations. The surprisingly rather low impact of the respiratory motion in atrial diastole may therefore result from the anatomic position of the LAA with relatively little influence of respiratory motion [31]. In addition, the patients were sedated during LAA closure which is known to cause shallow breathing [32].

Even though the achieved accuracy was in line with previously reported clinical demands, further improvement may be possible by using preprocedural data with higher spatial resolution images, e.g., CTA, and by ensuring a sufficient angular distance (35°–145°) between the two XR views used for 3D reconstruction as previously suggested [33]. Besides the consideration for 3D localization from two projections, the angular distance should also be considered for accurate co-registration with the XR system, and consequently accurate 3D localization on monoplane systems.

There are still some general limitations in this study. A major limitation of this study is the relatively small number of patients enrolled. Therefore, the results presented might be considered as indication of the relevance of motion-compensation with further validation in larger cohorts still needed. The co-registration as well as identification of the respiratory phase was done manually and using automated approaches such as, e.g., proposed earlier [15, 34, 35] might further increase the resulting accuracy. Further, the non-availability of XR runs in two angulations for the motion impact analysis and hence the projection of the marker displacement on the view axis might impact the absolute value of the differences between the analyzed data. Even though the absolute error may be underestimated for the non-motion synchronized data, the general trend and drawn conclusions should be correct. Further, the resulting error is likely intervention-specific. To draw a general conclusion regarding the relevance of motion synchronized pre- and periprocedural fusion, the accuracy has to be analyzed for different applications preferably in larger cohorts.

## Conclusion

The impact of motion synchronization on the 3D accuracy of image guidance intervention systems could be clearly shown. Even though residual error sources like patient repositioning accuracy, quality of co-registration, and system limitations like c-arm bending could not be fully considered, a high accuracy well below 5 mm could be proven even on monoplane systems.
